# Human Mast Cells From Adipose Tissue Target and Induce Apoptosis of Breast Cancer Cells

**DOI:** 10.3389/fimmu.2019.00138

**Published:** 2019-02-18

**Authors:** Jesse D. Plotkin, Michael G. Elias, Mohammad Fereydouni, Tracy R. Daniels-Wells, Anthony L. Dellinger, Manuel L. Penichet, Christopher L. Kepley

**Affiliations:** ^1^Department of Nanoscience, Nanobiology, Joint School of Nanoscience and Nanoengineering, University of North Carolina, Greensboro, NC, United States; ^2^Division of Surgical Oncology, Department of Surgery, David Geffen School of Medicine, University of California, Los Angeles, Los Angeles, CA, United States; ^3^Department of Microbiology, Immunology and Molecular Genetics, David Geffen School of Medicine, University of California, Los Angeles, Los Angeles, CA, United States; ^4^Jonsson Comprehensive Cancer Center, University of California, Los Angeles, Los Angeles, CA, United States; ^5^The Molecular Biology Institute, University of California, Los Angeles, Los Angeles, CA, United States; ^6^AIDS Institute, University of California, Los Angeles, Los Angeles, CA, United States; ^7^The California NanoSystems Institute, University of California, Los Angeles, Los Angeles, CA, United States

**Keywords:** mast cells, IgE, cancer, immunotherapy, breast cancer, HER2/*neu*

## Abstract

Mast cells (MC) are important immune sentinels found in most tissue and widely recognized for their role as mediators of Type I hypersensitivity. However, they also secrete anti-cancer mediators such as tumor necrosis factor alpha (TNF-α) and granulocyte-macrophage colony-stimulating factor (GM-CSF). The purpose of this study was to investigate adipose tissue as a new source of MC in quantities that could be used to study MC biology focusing on their ability to bind to and kill breast cancer cells. We tested several cell culture media previously demonstrated to induce MC differentiation. We report here the generation of functional human MC from adipose tissue. The adipose-derived mast cells (ADMC) are phenotypically and functionally similar to connective tissue expressing tryptase, chymase, c-kit, and FcεRI and capable of degranulating after cross-linking of FcεRI. The ADMC, sensitized with anti-HER2/*neu* IgE antibodies with human constant regions (trastuzumab IgE and/or C6MH3-B1 IgE), bound to and released MC mediators when incubated with HER2/*neu*-positive human breast cancer cells (SK-BR-3 and BT-474). Importantly, the HER2/*neu* IgE-sensitized ADMC induced breast cancer cell (SK-BR-3) death through apoptosis. Breast cancer cell apoptosis was observed after the addition of cell-free supernatants containing mediators released from FcεRI-challenged ADMC. Apoptosis was significantly reduced when TNF-α blocking antibodies were added to the media. Adipose tissue represents a source MC that could be used for multiple research purposes and potentially as a cell-mediated cancer immunotherapy through the expansion of autologous (or allogeneic) MC that can be targeted to tumors through IgE antibodies recognizing tumor specific antigens.

## Introduction

Mast cells (MC) are resident tissue immune cells that play an important role in innate and acquired immunity, but are most widely recognized in their role as regulators of Type I hypersensitivity (1, 2). Differences in MC phenotypes and functional responses between species have hampered progress in understanding their role in several disease processes (2–7). This incongruence has directed efforts toward obtaining sources of human MC that can be used to evaluate the role of these cells in basic mechanisms of disease without confounding differences between rodent and human systems (4, 5, 8). For example, MC can be obtained by culturing progenitor cells from cord blood, venous blood, fetal liver, bone marrow, and skin (8–12). However, variations in culture conditions and the resulting MC that are phenotypically and functionally immature still result in limitations that have hindered MC research. Thus, new sources of human MC are consistently needed.

One disease in which the role of MC has been investigated is cancer (13–15). It is controversial as to their role in this disease in light of contradictory findings between model systems and species and that studies in humans are solely correlative (i.e., an increase in MC numbers equates to poor prognosis (13, 16–18). Human MC contain several pro-inflammatory mediators, but are unique in their ability to pre-store and release potentially beneficial anti-cancer mediators. For example, human MC have pre-stored and releasable (through FcεRI engagement) tumor necrosis factor alpha (TNF-α) within their granules (2). Furthermore, human MC release granulocyte-macrophage colony-stimulating factor (GM-CSF) upon FcεRI stimulation (19, 20). Both TNF-α and GM-CSF have been used as anti-cancer agents (21, 22). Correlative studies in humans cannot address if the MC are affecting tumor growth; whether their presence enhances, inhibits, or are non-participating bystanders. Thus, developing ways to use MC to target tumors will aid researchers in determining the functional role of these cells in various tumors. In addition, harnessing anti-tumor agents from MC as a potential “Trojan Horse” may represent a new form of cancer cellular immunotherapy.

Human adipose tissue is a heterogeneous tissue containing the stroma-vascular (SVF) fraction that includes a large population of immune progenitor cells (23) and is a reservoir of functional MC progenitors in mice (24). We report here that large numbers of functional human MC can be expanded from human adipose tissue. The adipose-derived MC (ADMC) are phenotypically and functionally similar to connective tissue MC obtained from skin as assessed through MC-specific markers and IgE- and non-IgE-dependent mediator release assays. Importantly, ADMC sensitized with anti-HER2/*neu* IgE antibodies (Abs) are able to induce cell death in breast cancer cells overexpressing HER2/*neu*. Adipose tissue now provides researchers a new source of human MC that could be used for multiple research purposes and as a potential new strategy for cell-mediated cancer immunotherapy.

## Materials and Methods

### Consent Statement

Tissue procurement and IRB approval including patient consent were obtained from the Cooperative Human Tissue Network.

### Adipose Tissue Digestion

Skin and adipose tissue was obtained from patients undergoing cosmetic surgery. Adipose tissue was incubated with Hanks' balanced salt solution (HBSS), 1% fetal bovine serum, 0.04% sodium bicarbonate, 1% HEPES, 0.5% amphotericin B, 1% streptomycin/penicillin and 0.1% collagenase type 1A. Cells were placed into a 37°C orbital shaker for 1 h with constant agitation at 4 × g. The cell slurry was centrifuged at 360 × g for 15 min and adipocytes washed, suspended in medium (DMEM with 4.5 g/L glucose, 10% fetal bovine serum, 1% streptomycin/penicillin, 1% L-glutamine and 1% HEPES), and cultured for up to 7 days or until the stem cells were confluent before testing of MC-differentiating media below.

### Mast Cell Differentiation

Different cell culture media were tested for their ability to induce MC differentiation of the adipose cells using X-VIVO 15 or AIM-V (Lonza, Switzerland), plus 80 ng/ml SCF (Stemcell Technologies, Vancouver, BC) with or without non-specific (NS) psIgE (human myeloma IgE; a gift from Dr. Andrew Saxon, UCLA; 0.1 μg/ml). Conditioned MC media was produced using media used to culture primary human skin MC as described (12, 25). Briefly, skin MC cultures (>5 weeks) containing 80 ng/ml SCF in X-VIVO 15 were pelleted by centrifugation, supernatants removed, filtered through a 22 μm filter (Sigma-Aldrich, St. Louis, MO) to remove any cells, and added directly to the adipose stem cells (~15 ml per 75/mm^2^ flask). Approximately every seven to 10 days, viability was assessed by trypan blue exclusion and half of the media was collected and replaced with fresh media. Initial monitoring of MC differentiation was determined using toluidine blue staining of cytospins followed by further characterization as described below.

### Flow Cytometry

Flow cytometry was performed using a FACS Arial III (Becton Dickenson, Franklin Lakes, NJ). Briefly, mouse anti-human Abs to FcεRI, c-kit, FcγRI, FcγRII, FcγRIII (Santa Cruz, Dallas, TX), or mouse IgG isotype control MOPC (Sigma-Aldrich) were added for at least 1 h on ice, washed, and F(ab′)_2_-FITC-goat anti-mouse Abs (BD Biosciences, San Jose, CA) added for detection (26). All experiments were performed at least three times.

### Cytochemistry and Immunofluorescence

Immunochemistry was performed with mouse anti-human Abs to tryptase and chymase or NS mouse IgG isotype (negative) control as described (27, 28) but using Cy3-conjugated anti-mouse secondary Abs. For detection of ADMC-induced apoptosis of human breast cancer SK-BR-3 cells (ATCC, Manassas, VA), cell cytospins were incubated with 1 μg/ml Alexa Fluor^TM^ 488 dye (ThermoFisher Scientific, Walnut, CA) labeled mouse anti-human tryptase (1 μg/ml; for ADMC detection; green) along with Alexa Fluor^TM^ 647 labeled mouse anti-human Ab to the active form of caspase 3 (1 μg/ml; for SK-BR-3 detection; red) or Alexa Fluor^TM^ 647 labeled isotype control for the caspase 3 Ab. To quantify the percentage of caspase 3 positive cells observed on the cytospins a total of 200 cells were counted on each slide and the number of SK-BR-3 cells positive for caspase activation was compared to the number of those not stained for caspase 3 that were not MC.

### Gene Expression

RNA was extracted from ADMC using the Qiagen RNeasy Plus Mini kit (Qiagen, Germany). Reverse Transcriptase PCR (RT-PCR) was performed using the Qiagen OneStep RT-PCR kit using primers previously described to amplify short fragments of the β-actin, tryptase, chymase, c-KIT, and FcεR1α RNA (29). Cycling conditions were: 50°C for 30 min, 95°C for 15 min, followed by 35 cycles of 94°C for 45 s, 53–63°C for 45 s (according to primer T_m_), 72°C for 1 min and a final 10 min extension at 72°C.

### Anti-HER2/*neu* IgE Abs and Extracellular Domain of HER2/*neu* (ECD^HER2^)

The fully human anti-human HER2/*neu* IgE/kappa containing the variable regions of the human scFv C6MH3-B1 has been previously described (30). In addition, we also developed an anti-human HER2/*neu* IgE/kappa containing the variable regions of the humanized Ab trastuzumab (Herceptin®) by subcloning the variable regions of trastuzumab previously used in Ab-cytokine fusion proteins (31, 32) into the human epsilon/kappa expression vectors use to the develop the C6MH3-B1 IgE. The trastuzumab IgE and C6MH3-B1 IgE bind different epitopes of human HER2/*neu*. They are expressed in murine myeloma cells and the transfectomas grown in roller bottles for Ab production as described (30). The IgE Abs are purified from cell culture supernatants on an immunoaffinity column prepared with omalizumab (Xolair®) (Genentech, Inc. San Fransisco, CA, USA) (30). The extracellular domain of HER2/*neu* (ECD^HER2^) was produced as described previously (31). All proteins were quantified using the BCA Protein Assay (ThermoFisher Scientific).

### Degranulation and Cytokine Production From ADMC

To determine ADMC functional responses mediated through FcεRI, ADMC were incubated with 1 μg/ml of anti-FcεRI Abs or with 1 μg/ml anti-NP IgE for 1 h followed by NP-BSA. To determine ADMC functional responses mediated by non-IgE pathways, ADMC were incubated with 40 μg/ml Poly-L-Lysine (Sigma-Aldrich) or 10 μM A23187 (Sigma-Aldrich). Post-incubation, activation was performed for 30 min (to measure degranulation) or overnight (for cytokine analysis) and β-hexosaminidase release and TNF-α and GM-CSF production were measured as described (33–35). All experiments were performed in duplicate from four separate donors and significant differences (*p* < 0.05) determined using the Student *t-*test.

### HER2/*neu* IgE-Mediated Binding of ADMC to Breast Cancer Cells

To assess the ability of anti-HER2/*neu* IgE sensitized ADMC to bind to HER2/*neu* expressing SK-BR-3 breast cancer cells, confocal imaging was used on differentially labeled, live cells. The ADMC (1.5 × 10^5^) were sensitized with 1 μg/ml of anti-HER2/*neu* IgE Abs or NS psIgE followed by the addition of MitoTracker™ Green (500 nM; ThermoFisher Scientific). The ADMC were washed once in warm X-VIVO 15 and added to the adherent, human HER2/*neu*-positive SK-BR-3 cells that were pre-stained with MitoTracker™ Red (500 nM; ThermoFisher Scientific) in a live cell incubator affixed to a confocal microscope and images acquired over 6 h.

### Breast Cancer Cell-Induced Mediator Release From ADMC

ADMC were sensitized with or without 1 μg/ml of anti-HER2/*neu* IgE or NS psIgE as above and added to human breast cancer cells expressing high levels of HER2/neu SK-BR-3 or BT-474 (a gift from Dr. Hui-Wen Lo, Wake Forest University) cells for 1 h in 24 well plates. The ratio of MC to breast cancer cells varied from 1:10 to 10:1 ADMC to breast cancer cells and mediators assessed in the supernatants. In some experiments anti-HER2/*neu* IgE sensitized ADMC challenged with ECD^HER2^ or heat-inactivated serum from patients with HER/*neu* positive breast cancer (Cureline, Brisbane, CA; Table 1).

**Table 1:** HER2/*neu* positive breast cancer patient serum.

**Serum**	**Diagnosis**	**Age**	**Pathological diagnosis**	**Grade**	**TNM staging (T)**	**TNM staging (N)**	**TNM staging (M)**	**Stage**	**HER2/*neu* status**	**Treatment history**
P1	Breast carcinoma	64	Infiltrative ductal carcinoma	G3	T2	N1	M0	IIB	2+	None (treatment-naive)
P2	Breast carcinoma	35	Infiltrative introductal carcinoma	G1	T1	N0	M0	IA	3+	None (treatment-naive)

### HER2/*neu* IgE-Mediated Killing of Breast Cancer Cells by ADMC and Supernatants From Activated ADMC

Three different methods were used to assess the ability of anti-HER2/*neu* IgE sensitized ADMC to induce cell death of HER2/*neu* expressing breast cancer cells. First, ADMC (1.5 × 10^5^) were sensitized with 1 μg/ml of anti-HER2/*neu* IgE or psIgE for 2 h. Breast cancer cells (5 × 10^4^) on coverslips were labeled with 2 μM MitoTracker™ Green (ThermoFisher Scientific) for 1 h. The washed ADMC were labeled with CellTracker^TM^ Deep Red (which stains the cells reddish/purple under confocal; 2 μM) for 1 h, washed, and added to SK-BR-3 in medium containing 25 μg/ml of propidium iodide (PI; which stains the cells red) used to detect dead cells (36) and PI intensity measured over time. Second, SK-BR-3 were plated and incubated with Cellevent^TM^ Caspase 3/7 Green (to detect activated caspase-3/7 in apoptotic cells; ThermoFisher Scientific) for 1 h according to the manufacturers protocol. ADMC, treated with MitoTracker^TM^ Red (1 μg/ml), were added to the washed SK-BR-3 cells and incubated for up to 4 days. Third, cytospins of cells from separate experiments were made and used for immunofluorescence detection of apoptosis. Briefly, cytospins were fixed in methanol and incubated with Alexa Fluor^TM^ 488 dye (ThermoFisher Scientific) labeled mouse anti-human tryptase (1 μg/ml; for ADMC detection; green, for co-cultures) along with Alexa Fluor^TM^ 647 labeled mouse anti-human active caspase 3 (BD Biosciences, 1 μg/ml; for SK-BR-3 detection; red) Alexa Fluor^TM^ 647 labeled isotype Abs were used as a control for the caspase 3 Ab.

In separate experiments, cell free supernatants from optimally activated ADMC (1.3 × 10^6^) by an anti-FcεRI Ab (1 μg/ml for 24 h; 60–70% release) were directly added to MitoTracker™ Green-labeled SK-BR-3 cells (5 × 10^4^). In some experiments, an anti-human TNF-α Ab (5 μg/ml) was added to the supernatants to block TNF-α activity. Cell death was monitored over time through the quantification caspase 3/7-positive cells (>200) counted at the end of each experiment to obtain percentages. All confocal/live cell experiments were performed on three separate ADMC donors and significance (*p* < 0.05) tested using the Student's *t-*test.

## Results

### Phenotypic Characterization of ADMC

**Figure 1:**
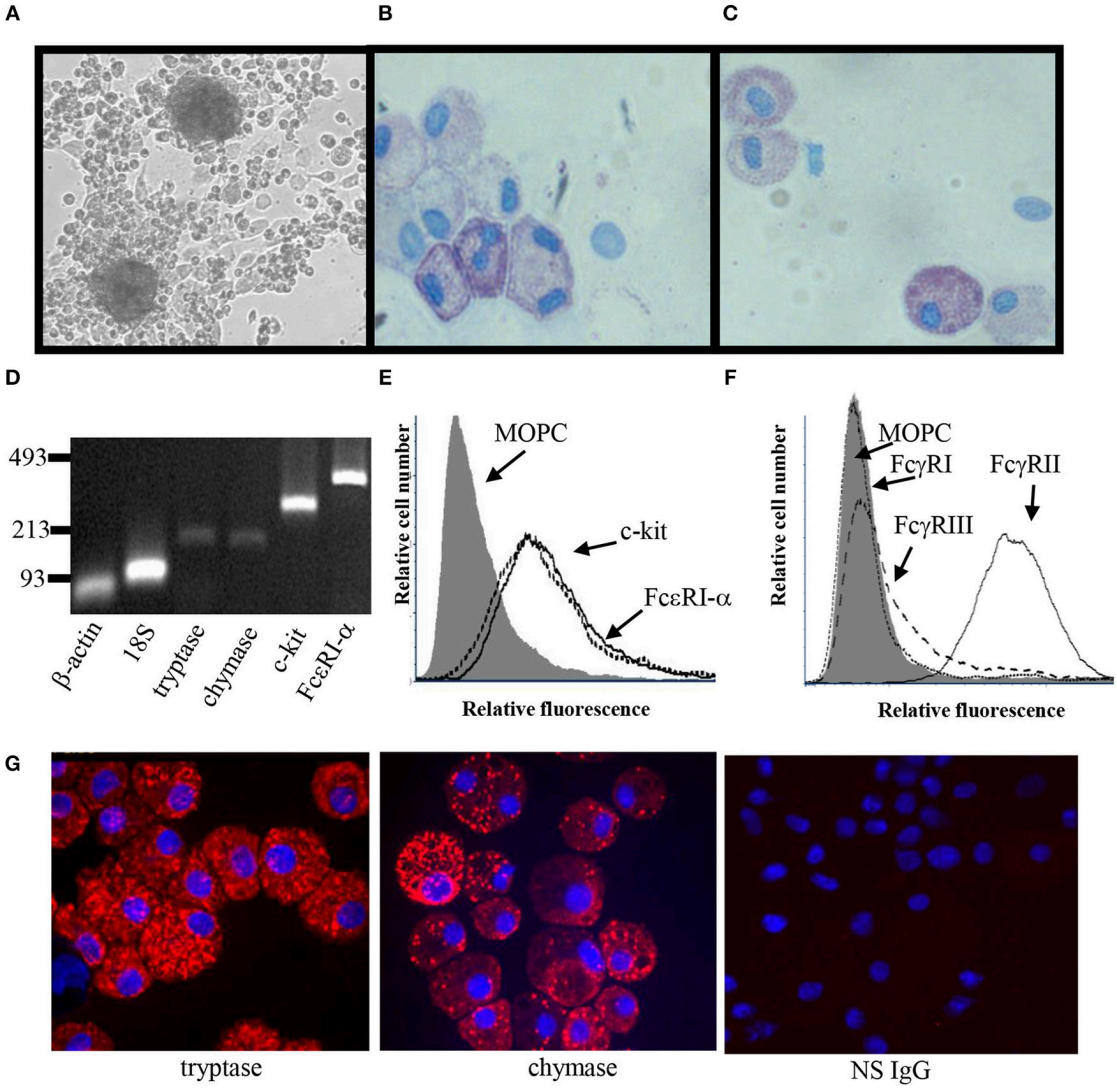
Phenotypic characterization of ADMC. **(A)** Light microscopy of ADMC. ADMC cultures demonstrating large cell/tissue clumps from which the MC differentiate (20 × magnification). Cytospins of adipose-derived **(B)** or skin MC **(C)** were stained with toluidine blue. **(D)** Gene expression was measured using RT-PCR on total RNA. β-actin and 18S ribosomal subunit primers were controls (Ladder: bp). **(E)** Surface expression of MC-specific markers by flow cytometry. ADMC were incubated with mouse anti-c-Kit/CD117 (dashed line), FcεRIα chain (solid line), or isotype control mouse IgG (gray) for 2 h on ice, washed, and F(ab′)_2_-FITC-goat anti-mouse added for 1 h. **(F)** Fcγ receptor expression on ADMC. ADMC were incubated with mouse anti human FcγRI (dotted), FcγRII (solid line), FcγRIII (dashed), or isotype control mouse IgG (gray) for 2 h on ice, washed, and FITC-labeled anti-mouse F(ab′)_2_ added for 1 h. **(G)** Immunohistochemistry of ADMC with MC-specific markers. Anti-tryptase, anti-chymase, or NS IgG Ab were incubated overnight on cytospin cells, washed and incubated with Cy3-secondary Abs and Hoechst dye (blue nuclei) and visualized using confocal microscopy. Figures are representative of cells derived from three different human subjects.

Several culture conditions were tested for their ability to induce MC differentiation (35, 37). The conditioned media from skin-derived human MC cultures was found to be optimal for ADMC differentiation (Table 2). In conditioned medium, ADMC were observed to emerge from large clumps of cells or tissue as shown in Figure 1A. After 3–4 weeks of culture, mature MC (>90% viable) were observed as demonstrated by the classical spherical, highly granulated morphology (Figure 1B) characteristic of skin-derived MC (Figure 1C). In addition, the ADMC were positive for messenger RNA to the two major MC proteases, tryptase, and chymase (Figure 1D). Furthermore, the ADMC expressed surface markers for tissue MC including FcεRI and the receptor for SCF, c-kit (Figure 1E). As previously reported with skin MC (26), ADMC express FcγRII and not FcγRI or FcγRIII (Figure 1F). As seen in Figure 1G both tryptase and chymase protein was detected using immunohistochemistry. Thus, adipose tissue has MC progenitors that can be differentiated into MC that are phenotypically similar to human connective tissue (MC_TC_) (38) based on these characteristics. Representative numbers of ADMC obtained from surgical specimens are shown in Table 3.

**Table 2:** Media used for MC differentiation.

**Media**	**Media additions**		**MC numbers**
	**80 ng/ml**	**0.1 μg/ml**	
AIM-V	SCF		+
	SCF	IgE	+
X-VIVO 15	SCF		+
	SCF	IgE	++
Conditioned media	None added		+++

**Table 3:** Representative MC numbers from skin vs. liposuction tissue using conditioned medium.

**Adipose source**	**Starting grams or ml**	**MC numbers at 8 weeks**	**Cells per gram/ml**
Skin resection; BF	50 g	9.9 × 10^6^	2.0 × 10^5^
Skin resection; BF	27 g	8.6 × 10^6^	3.2 × 10^5^
Skin resection; WF	23 g	7.9 × 10^6^	3.4 × 10^5^
Skin resection; WF	32 g	2.4 × 10^7^	7.5 × 10^5^
Skin resection; WF	38 g	8.9 × 10^7^	7.6 × 10^5^
		Average/g	4.8 × 10^5^
Lipo; WF	150 ml	4.1 × 10^7^	2.7 × 10^5^
Lipo; WF	500 ml	3.2 × 10^8^	6.4 × 10^5^
Lipo; WF	750 ml	5.6 × 10^8^	7.5 × 10^5^
Lipo; WF	200 ml	7.1 × 10^7^	3.6 × 10^5^
		Average/ml	5.1 × 10^5^

*BF, Black female; WF, White female*.

### Functional Characterization of ADMC

**Figure 2:**
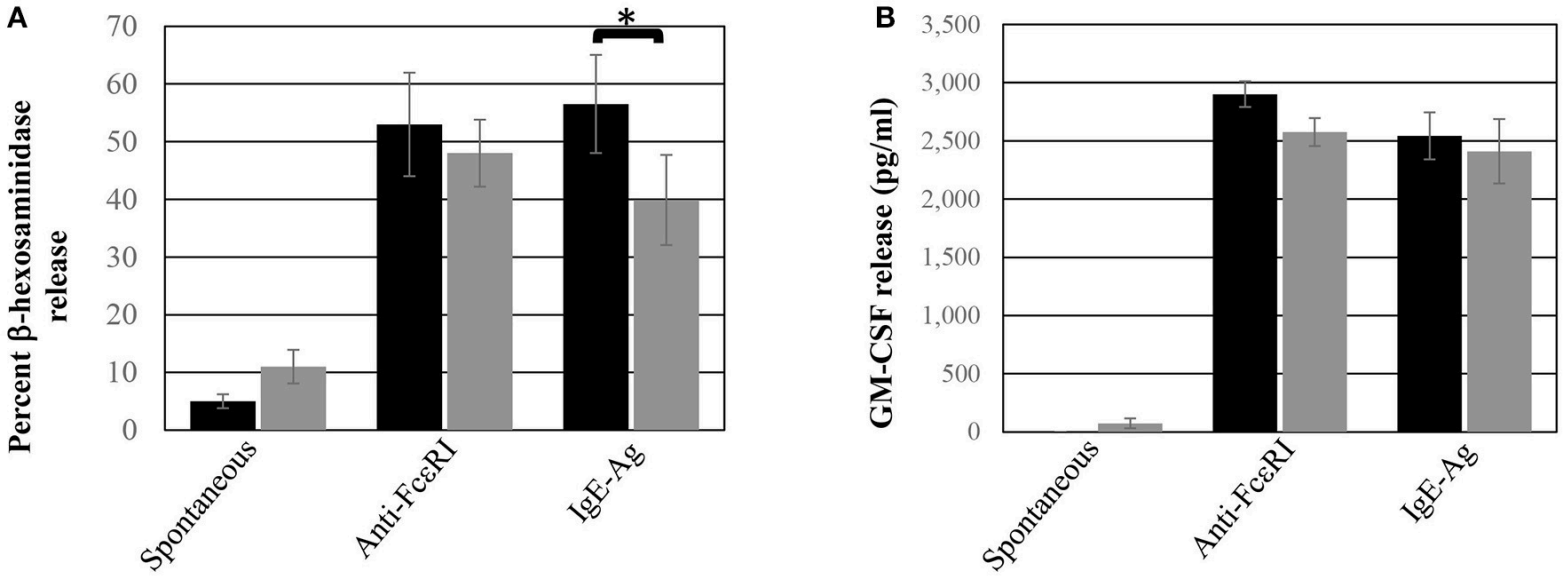
ADMC functional response. Human skin MC (black box) or ADMC (gray box; 10^6^) were challenged with or without (spontaneous release) 1 μg/ml anti-FcεRI Abs or anti-NP IgE + antigen (IgE-Ag) and degranulation **(A)** or GM-CSF production **(B)** assessed in the supernatants. Error bars represent ± SD. *p < 0.05 comparing skin MC vs. ADMC release. Figure is representative of cells derived from two different donors.

The functional response of ADMC was compared to skin-derived MC. As seen in Figure 2, ADMC degranulated (Figure 2A) and produced cytokines (Figure 2B) in response to FcεRI engagement. Cytokine release by ADMC and skin MC was similar in response to FcεRI-dependent stimuli averaging 2,850 and 2,600 pg/ml of GM-CSF in skin MC and ADMC, respectively. A similar degranulatory response with ADMC was observed using non-FcεRI-dependent stimuli Poly-L-Lysine and A23187 (Supplemental Figure 1). Taken together, the ADMC are functionally similar to skin-derived MC in response to FcεRI-dependent and FcεRI-independent stimuli.

### Anti-HER2/*neu* IgE Mediates ADMC Binding to SK-BR-3 Breast Cancer Cells

**Figure 3:**
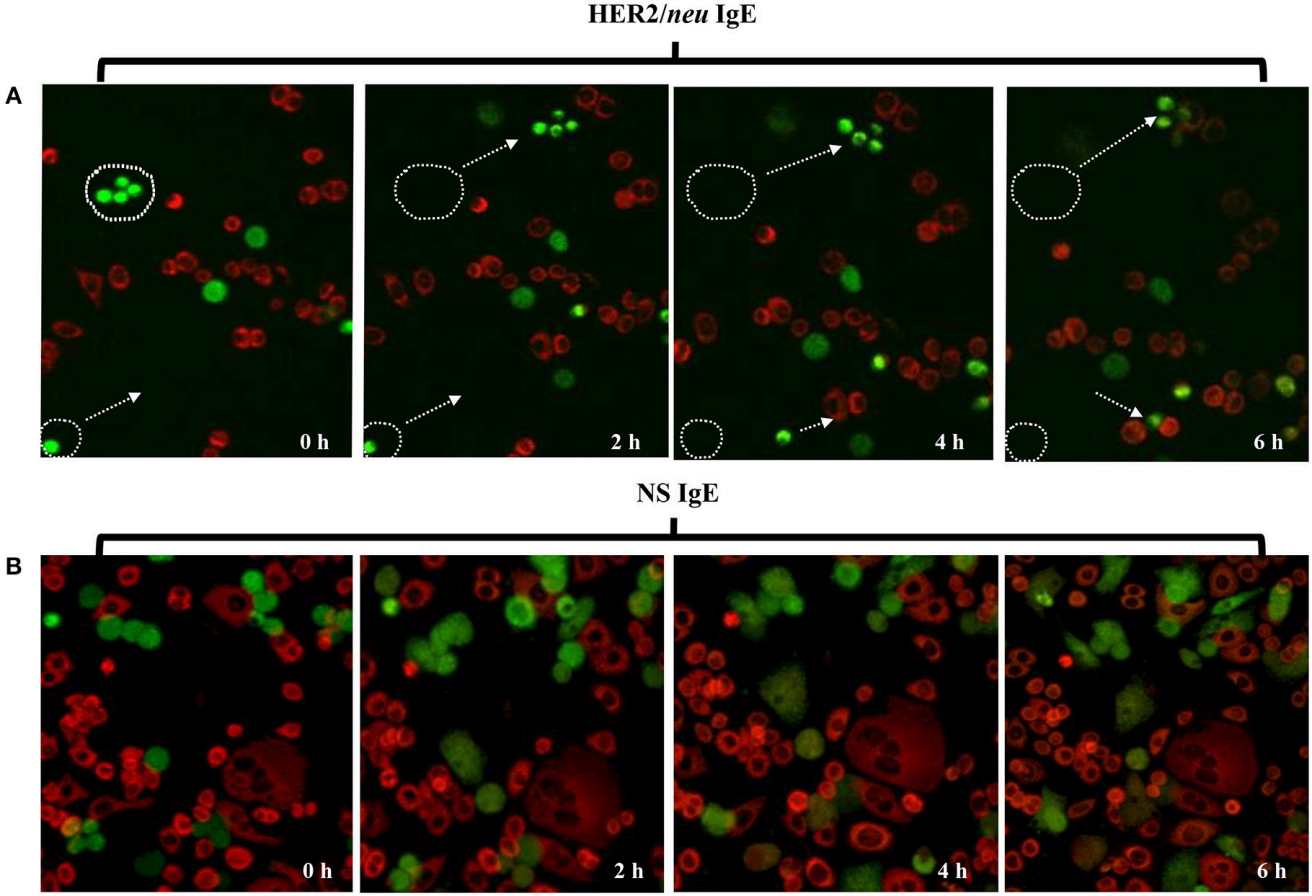
Time lapse, confocal microscopy of ADMC binding to breast cancer cells. ADMC (10^5^-10^6^) were sensitized with 1 μg/ml of trastuzumab IgE (**A**, 20X) or NS IgE (**B**, 40X) followed by MitoTracker™ Green. The MitoTracker™ Green-loaded ADMC were added to adherent SK-BR-3 (10^5^-10^6^) that had been pre-stained with MitoTracker™ Red and time lapse video taken over 6 h. The white circular boundaries and arrows represent starting point and tracking of ADMC (green) at time 0 to SK-BR-3 (red) binding over the 6 h. 20X magnification was used to capture the cellular tracking (start and stop) that can be observed in accompanying video.

The ability of ADMC sensitized with the anti-HER2/*neu* IgE to bind HER2/*neu*–positive SK-BR-3 breast cancer cells was investigated. As seen in Figure 3A, the ADMC sensitized with the anti-HER2/*neu* IgE (green) bound to HER2/*neu*-positive SK-BR-3 breast cancer cells (red) as demonstrated in the time lapse pictures and video (Supplemental Video 1). However, ADMC sensitized with a NS IgE did not target or bind to the SK-BR-3 cells (Figure 3B). These results demonstrate that the anti-HER2/*neu*-IgE mediates the interaction between ADMC and HER2/*neu*-positive breast cancer cells.

### Anti-HER2/*neu* IgE-Sensitized ADMC Become Activated Through FcεRI Upon HER2/*neu*—Positive Breast Cancer Cell Binding

**Figure 4:**
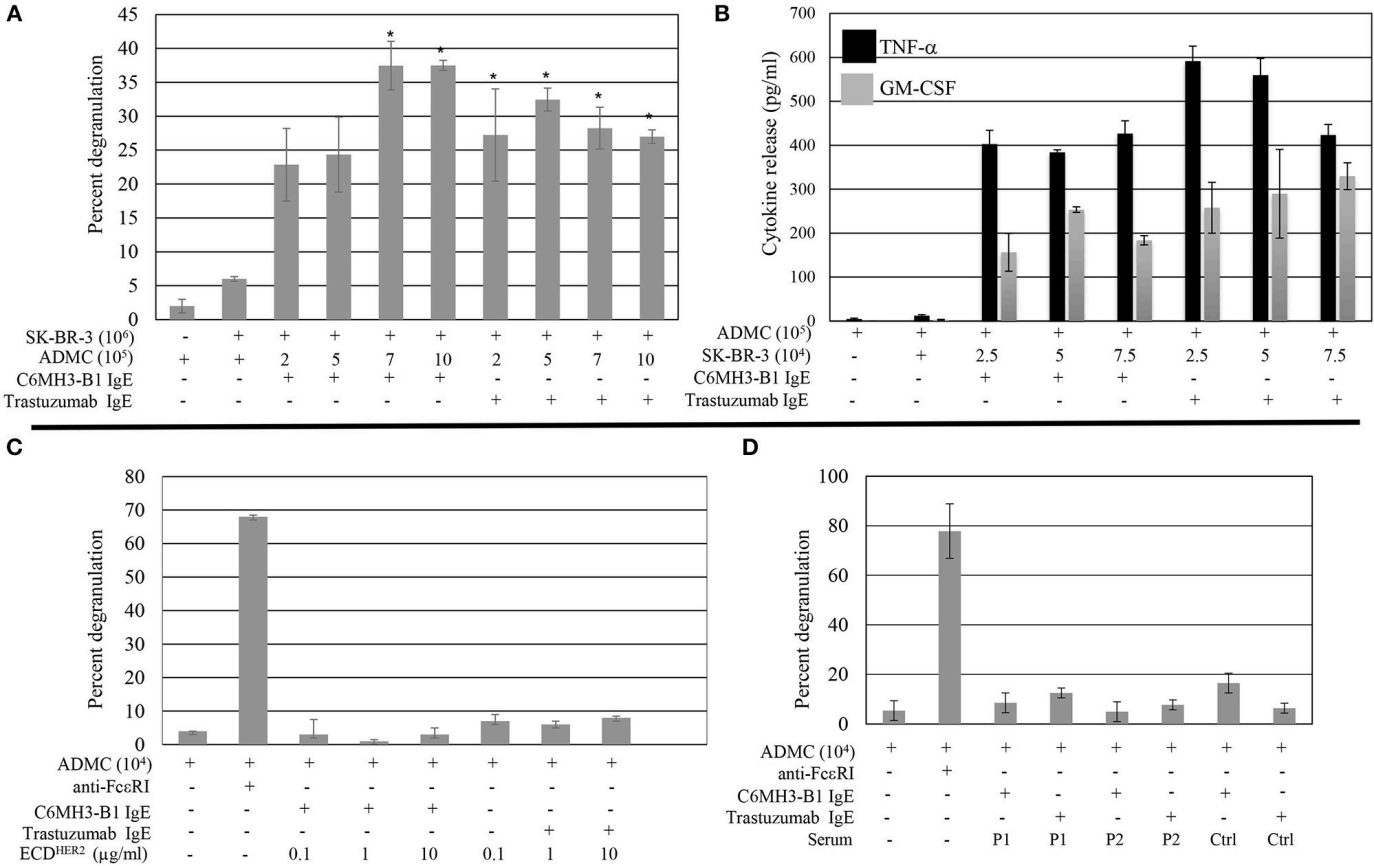
Breast cancer cell-induced ADMC mediator release. ADMC were sensitized with 1 μg/ml anti-HER/*neu* IgE (clone C6MH3-B1 IgE or trastuzumab IgE), washed, and incubated with SK-BR-3 cells and degranulation **(A)** or cytokine release **(B)** assessed. Data are from a single experiment representative of experiments performed on cells derived from four separate donors. Error bars represent ± SD. *p < 0.05 Compared with non-IgE (spontaneous) release. All values in **(B)** are significant compared to spontaneous release. **(C)** ECD^HER2^ does not induce ADMC mediator release. ADMC were sensitized with 1 μg/ml anti-HER/*neu* IgE as in **(A)**, washed, and incubated with ECD^HER2^ and mediator release assessed. As a control, optimal concentrations of 1 μg/ml anti-FcεRI Ab were tested in parallel. Each condition was tested in triplicate and is representative from two separate ADMC donors. Error bars represent ±SD. **(D)** Sera from HER2/*neu* positive breast cancer patients does not induce ADMC degranulation. Heat inactivated sera from two separate HER2/*neu* positive breast cancer patients (P1 and P2; see Table 1) or normal control serum (Ctrl) was used to challenge anti-HER/*neu* IgE (C6MH3-B1 IgE or trastuzumab IgE) sensitized ADMC and β-hexosaminidase release measured as described. Background levels of β-hexosaminidase naturally found in the sera was subtracted from values. Experiment is representative of two separate ADMC preparations each done in duplicate. Error bars represent ± SD.

ADMC must release their mediators upon FcεRI challenge at the site of the tumor to be effective anti-tumor agents. Thus, the ability of ADMC sensitized with the anti-HER2/*neu* IgE to degranulate in the presence of breast cancer cells was investigated. ADMC were sensitized with one of two anti-HER2/*neu* IgE Abs recognizing different epitopes (trastuzumab IgE or C6MH3-B1 IgE). Varying ADMC cell numbers were incubated with SK-BR-3 breast cancer cells and mediator release assessed in the medium. As seen in Figure 4, ADMC sensitized with anti-HER2/*neu* IgE induced significant (*p* < 0.05) mediator release through FcεRI when co-incubated with the HER2/*neu*-positive SK-BR-3 breast cancer cells. The ADMC degranulated to release pre-stored mediators (Figure 4A), as well as newly formed mediators TNF-α and GM-CSF (Figure 4B). Another HER2/*neu*-positive breast cancer cell line, BT-474, also induced degranulation and cytokine production optimally at a ratio of 1:2 (degranulation) and 1:0.5 (cytokine release) ADMC:BT-474 (Supplemental Figure 2).

The above results suggest the possibility of using ADMC armed with IgE Abs can trigger degranulation in the presence of HER2/*neu* expressing cancer cells and thus, the potential of using this strategy for cancer therapy via the release of MC mediators. However, a potential concern of the systemic administration of ADMC sensitized with an anti-HER2/*neu* IgE is the possible induction of a systemic anaphylactic reaction as patients with HER2/*neu* breast cancer can have elevated levels of circulating ECD^HER2^ in the blood (39, 40). The IgE Abs are not expected to induce FcεRI cross-linking when complexed with soluble antigen (ECD^HER2^), given the mono-epitopic nature of this interaction and the fact that ECD^HER2^ does not form homodimers in solution (41, 42). To address this concern, the ability of ECD^HER2^ to induce FcεRI-mediator release was examined. As described previously (30), ECD^HER2^ in the presence of the anti-HER2/*neu* IgE Abs did not induce degranulation, while anti-FcεRI Abs induced release (Figure 4C). Furthermore, serum from two separate HER2/*neu* positive breast cancer patients did not induce ADMC degranulation (Figure 4D). These results suggest that the anti-HER2/*neu* IgE-sensitized ADMC will not induce a systemic anaphylactic response *in vivo* and will only release mediators upon encountering HER2/*neu* on breast cancer cells.

### IgE Sensitized ADMC Induce SK-BR-3 Cell Death

**Figure 5:**
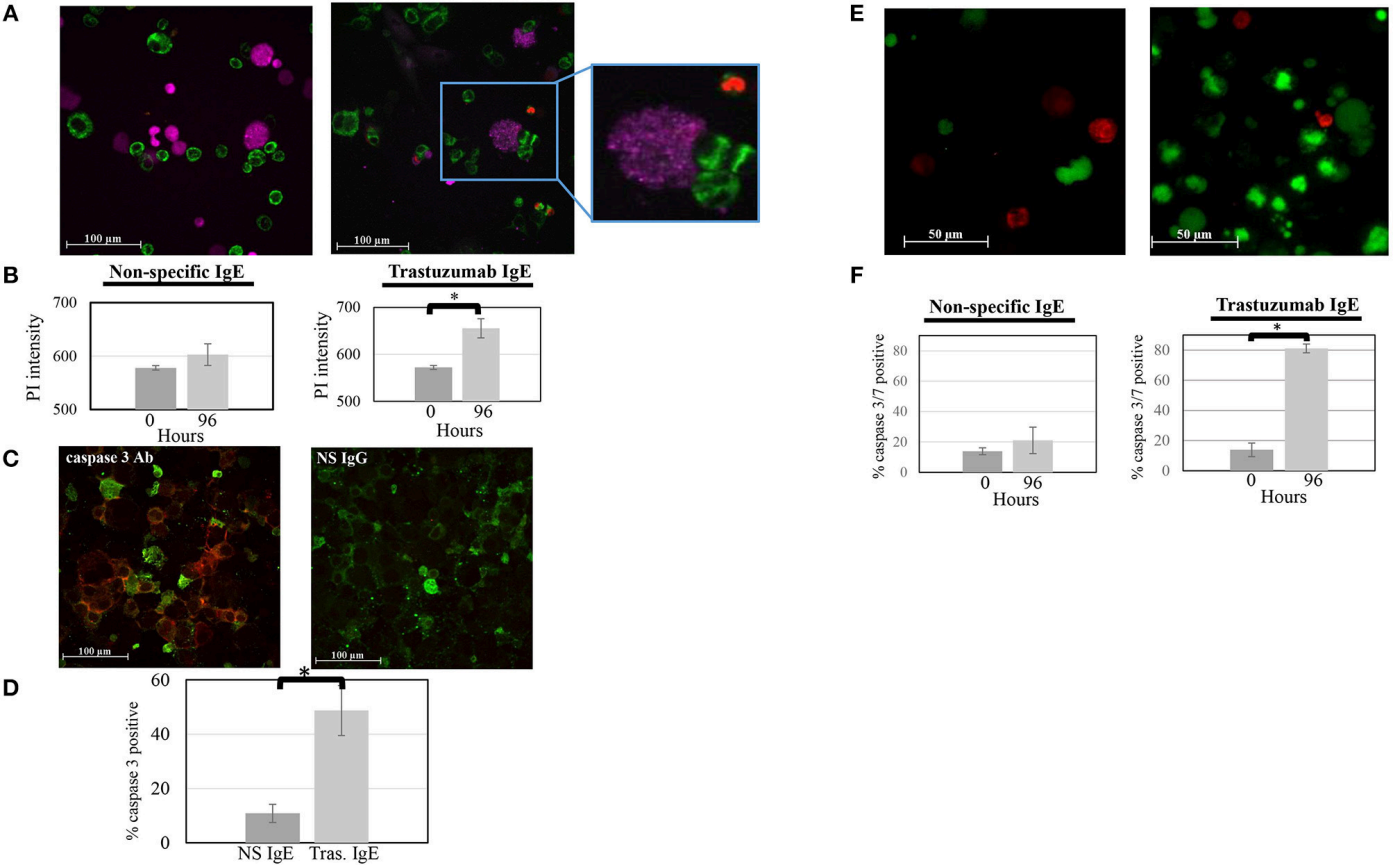
ADMC killing of human breast cancer cells measured as evidence by the uptake of PI. **(A)** CellTracker™-red labeled ADMC (7.5 × 10^4^; shown here as purple cells) were sensitized with 1 μg/ml anti-HER2/*neu* IgE (clone trastuzumab), washed, and incubated with MitoTracker™-green-stained SK-BR-3 (10^5^) in culture medium containing PI and images taken before (**A**; left) and after (**A**; right) 96 h. The call out box shows a representative ADMC being activated through loss of granularity over time. (Mag 20x). **(B)** Quantification of overall PI fluorescence before and after incubation. The percent of PI-positive cells was counted in culture. The *p = 0.0003 SK-BR-3 cell death at day 4 compared to day 0 when ADMC where sensitized with anti-HER2/*neu* IgE. No cell death was observed with the NS IgE. **(C)** ADMC-induced breast cancer cell apoptosis. Anti-HER2/*neu* IgE-sensitized ADMC (7.5 × 10^4^) were incubated with SK-BR-3 (1 × 10^5^) for 72 h, cytospins made, fixed, and incubated with Alexa Fluor^TM^ 488 labeled, mouse anti-human tryptase (left; green) along with Alexa Fluor^TM^ 647 labeled, mouse anti-human caspase 3 (red) or Alexa Fluor^TM^ 647 labeled, isotype control IgG for caspase 3 Ab (right). **(D)** Quantification of overall Alexa Fluor^TM^ 647 fluorescence before and after incubation. *p = 0.002 SK-BR-3 apoptosis comparing psIgE and anti-HER2/*neu* IgE-sensitized cells at day 4 from three experiments. **(E)** ADMC killing of human breast cancer cells measured by caspase 3/7 activation Mitotracker™-red labeled ADMC (7.5 × 10^4^; shown here as red cells) were sensitized with 1 μg/ml anti-HER2/*neu* IgE (clone trastuzumab), washed, and incubated with caspase 3/7 green-labeled SK-BR-3 (10^5^ and images taken before (**A**, left) and after (**A**; right) 96 h. **(F)** Quantification of overall caspase 3/7 fluorescence before and after incubation. The percent of caspase 3/7-positive cells was counted in culture. *p = 0.004 SK-BR-3 cell death at day 4 compared to day 0 when ADMC where sensitized with anti-HER2/*neu* IgE. No SK-BR-3 cell death was observed with the NS IgE.

The ability of ADMC to induce breast cancer cell death was investigated. ADMC sensitized with the anti-HER2/*neu* IgE were added to SK-BR-3 cells in medium containing PI to discriminate dead cells from live cells (36). As seen in Figure 5A, binding of anti-HER2/*neu*–sensitized (trastuzumab IgE) ADMC to SK-BR-3 cells induced significant cell death of the breast cancer cells as assessed by the uptake and visualization (red) of the PI in the SK-BR-3 cells but not the ADMC. Quantification of the PI signal in Figure 5B demonstrated significant breast cancer cell killing after 4 days (*p* = 0.0003). Sensitization of the ADMC with NS IgE did not result in significant SK-BR-3 cell death. Similarly, anti-HER2/*neu* IgE C6MH3-B1 sensitized ADMC induced significant (*p* = 0.032) SK-BR-3 cell death (data not shown). In addition, ADMC added to the SK-BR-3 over 4 days revealed significant (*p* = 0.003) breast cancer cell death, but not ADMC death (anti-tryptase labeled), as indicated by immunostaining of the SK-BR-3 with an Ab specific for the apoptotic enzyme caspase 3 (Figures 5C,D). Lastly, a significant (*p* = 0.0004) increase in caspase 3/7-positive breast cancer cells was confirmed at day 4 when anti-HER2/*neu*–sensitized ADMC were co-incubated (Figures 5E,F). The tumor cell specificity of the responses was verified as the NS isotype control IgE did not affect breast cancer cell viability. These experiments indicate ADMC binding to SK-BR-3 results in ADMC activation through FcεRI capable of inducing significant SK-BR-3 cell death.

### Mediators Released From ADMC Through FcεRI Induce SK-BR-3 Cell Death

**Figure 6:**
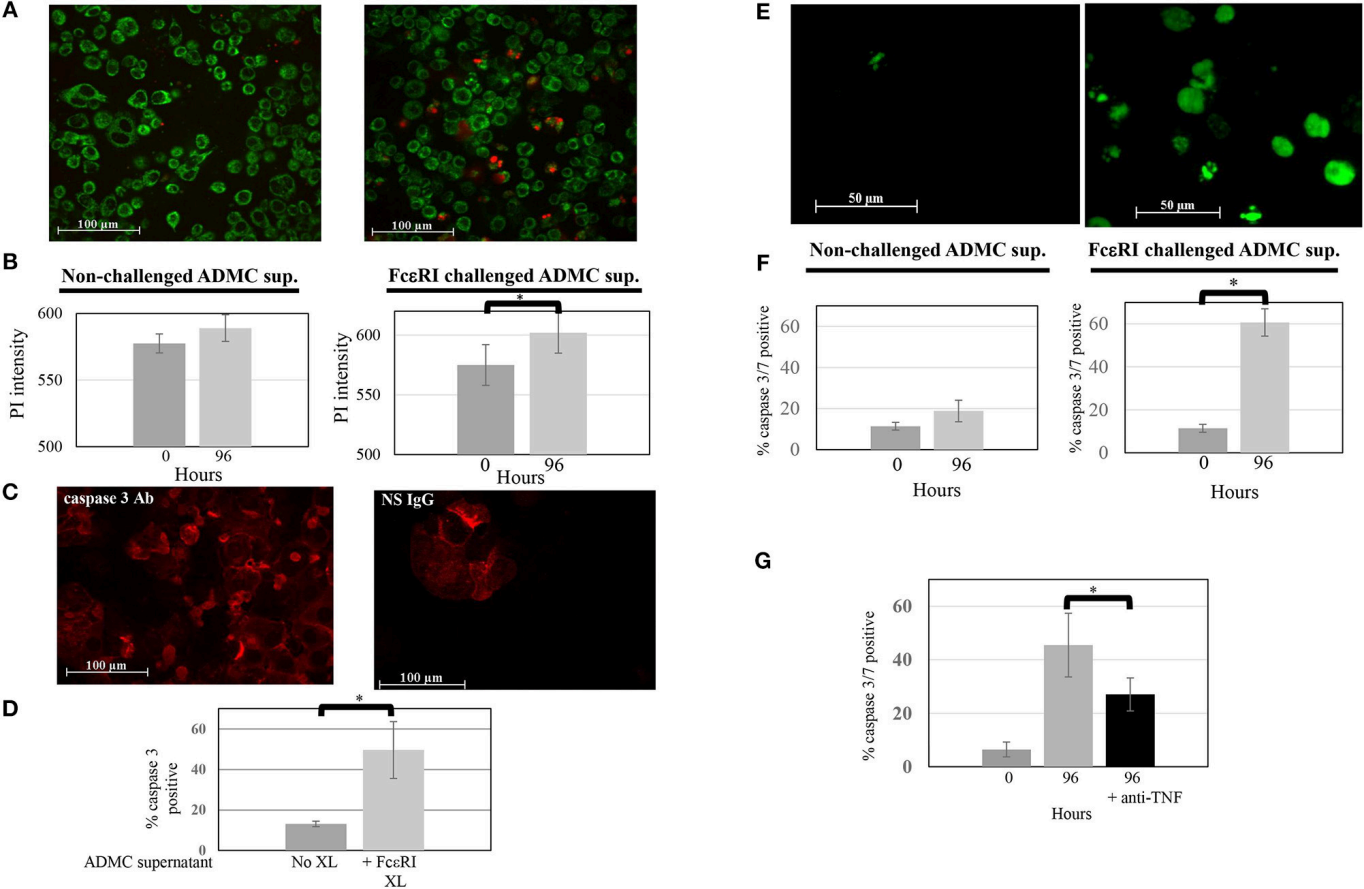
Mediators from FcεRI-challenged ADMC induce SK-BR-3 cell killing. **(A)** ADMC (1.3 × 10^6^) were challenged with optimal concentrations of anti-FcεRI stimuli (70% release) for 24 h and supernatants (XL media; no cells) from these ADMC were incubated with the MitoTracker™ green-stained SK-BR-3 (10^5^) in culture medium containing optimal concentrations of PI and images taken before (left) and after (right) 96 h. **(B)** Quantification of overall PI fluorescence before and after incubation. The increased number of red cells indicates breast cancer cell death as indicated by the PI (red) and quantified in showing overall PI fluorescence before and after incubation. Graph represents average PI intensity from two separate experiments (±SD; *p = 0.0008). **(C)** Mediators from FcεRI-challenged ADMC induce human breast cancer cell apoptosis. The same media from anti-FcεRI challenged ADMC were incubated with SK-BR-3 (10^5^) for 72 h, cytospins prepared, fixed, and incubated with Alexa Fluor 647 labeled, anti-human caspase 3 (left) or Alexa Fluor 647 labeled, isotype control Ab for caspase 3 (right). Representative panels are shown. **(D)** Quantification of overall Alexa Fluor^TM^ 647 fluorescence before and after incubation with supernatants from FcεRI activated ADMC. *p = 0.01 SK-BR-3 apoptosis comparing anti-HER2/*neu* IgE-sensitized cells at day 0 vs. day 4 from three experiments. **(E)** Mediators from FcεRI-challenged ADMC induce SK-BR-3 cell killing measured by caspase 3/7. ADMC (1.8 × 10^6^) were challenged with optimal concentrations of anti-FcεRI stimuli (63% release) for 24 h and supernatants (no cells) from these ADMC were incubated with SK-BR-3 (10^5^) and caspase 3/7 green images taken before (left) and after (right) 96 h. The increased number of green cells indicates breast cancer cell death as indicated by the caspase 3/7 and quantified by counting live vs. dead cells before and after incubation. **(F)** Graph represents average percentage of cells from two separate experiments (±SD; *p = 0.002). **(G)** Blocking TNF-α significantly reduces SK-BR-3 cell death. SK-BR-3 were treated and quantified as in **(C)** except anti-TNF-α Ab were added during the incubation time. *p = 0.028 Decrease in SK-BR-3 cell death when anti-TNF-α Ab are added to the supernatants from anti-FcεRI stimulated ADMC.

As shown above, ADMC produce mediators that induce significant breast cancer cell death upon FcεRI cross-linking using a tumor-targed IgE. The ability of the mediators obtained from FcεRI-activated ADMC alone to induce SK-BR-3 cell death was then examined. As seen in Figures 6A,B, medium alone (not containing ADMC) from optimally activated ADMC incubated with SK-BR-3 cells induced significant (*p* = 0.009) SK-BR-3 cell death when incubated for 4 days. Further, when the media from optimally activated FcεRI ADMC were added to the SK-BR-3, there was a significant (*p* = 0.01) increase of apoptotic cells as evidenced by the increase in active caspase 3 (Figures 6C,D) indicating cell death of the breast cancer cells as in Figure 5. A significant (*p* = 0.0002) increase in activated caspase 3/7-positive breast cancer cells was confirmed at day 4 when SK-BR-3 cells were incubated with supernatants from ADMC activated through FcεRI (Figures 6E,F). Blocking TNF-α activity significantly prevented SK-BR-3 cell death (Figure 6G).

## Discussion

Here we report that functional MC can be differentiated from adipose tissue obtained from human subjects undergoing cosmetic surgery procedures. This research discovery is notable as there is an ever-present need for new sources of human MC for research, given the differences between human and rodent MC phenotypes and functional responses (5–7). This incongruence has led to confusion and inconsistent findings in the field of MC biology and allergic mechanisms (8, 43, 44), especially in Fc receptor expression and function (26). While a plethora of human “mast cell” lines exist, each is wrought with phenotypic and functional anomalies compared to primary human MC (8). Primary human MC can be obtained from cord blood (45, 46), bone marrow (45), fetal liver (47), peripheral blood (48), and human tissue (e.g., skin) (11). For autologous applications, MC can be obtained from CD34+ hematopoietic progenitor cells in the blood (49), but not in sufficient numbers for most applications. For example, the total MC number generated from 1.0 × 10^8^ lymphocytapheresis or peripheral blood mononuclear cells averaged 2.5 × 10^6^ and 2.4 × 10^6^, respectively (50). Large numbers of enriched CD34+ cells can also be obtained commercially to increase the quantities of subsequent MC following GM-CSF injection, apheresis, and subsequent positive selection with magnetic beads has been described (48). However, given the various protocols for differentiation the MC obtained from these methods are not fully mature and functional. In this report, approximately 5.1 × 10^5^ ADMC were obtained per ml of liposuction compared to 4.8 × 10^5^ MC per gram of skin. Thus, ADMC can be utilized as a relatively rapid, more cost effective, and efficient method for studying MC biology and function. Current efforts are focused on identifying the molecule(s) in the conditioned media that are responsible for the ADMC differentiation and maturation.

The role of MC in cancer is controversial as to whether they are beneficial, harmful, or innocuous and is dependent on the tumor type and location within the tumor in humans and animal models (16, 18, 51–53). Animal models, mostly MC-deficient mice, have suggested that MC and their mediators play a pro-tumorigenic role (52). Yet, MC-deficient mouse models have paradoxically indicated that in certain tumors, and even in the same models, MC appear to play a protective role (52, 54). These contradictory results might reflect differences in the stage, incongruences between animal models (i.e., MC knockout through *kit* mutation vs. *Cre* mutation) and/or rodent MC lines vs. human MC, grade, and subtypes of tumor; as well as the different methods to identify MC. While in certain human cancers the presence of MC is associated with poor prognosis, in other malignancies, such as breast and colorectal cancer, the presence of MC has been associated with a favorable clinical prognosis depending on their location (55–58). Currently, multiple questions remain as to the nature of the role of MC in cancer pathogenesis.

Human MC are unique in that they have pre-stored TNF-α stores within their granules (59, 60). Furthermore, human MC release copious amounts (2,500–4,000 pg/ml from 10^5^ cells) of GM-CSF upon FcεRI stimulation (19, 20). Indeed, the above blocking experiments suggest TNF-α activity is the major component in FcεRI-activated ADMC supernatants that induces SK-BR-3 apoptosis (Figure 6G). TNF-α is an anti-cancer agent shown to suppress tumor cell proliferation, induce tumor regression, and used as an adjuvant that enhances the anti-cancer effect of chemotherapeutic agents (61–63). GM-CSF is also being investigated as an anti-breast cancer therapeutic, including its use in combination strategies with other immunotherapies (21, 64–67). There are over 50 clinical trials completed or underway examining the beneficial clinical effects of GM-CSF (www.clinicaltrials.gov). In addition to GM-CSF and TNF-α, MC also store and release several other potential anti-tumor mediators including reactive oxygen species (ROS), prostaglandin D2 (PGD2), interleukin-9 (IL-9), and heparin (2, 13). In one study cord blood-derived MC and eosinophils, sensitized with an anti-CD20 IgE, were shown to kill CD20-positive cancer cells (68). Thus, it may be possible that even in cases where MC may act favoring the tumors in certain cases through a controlled release of certain agents, they may have anti-tumor activity upon an IgE-mediated strong and immediate release of their granular content. Given that these MC mediators may have unwanted side effects, further *in vivo* studies are needed to address this topic.

There are 21 FDA approved Abs on the market to treat various cancers (69). While all are of the human IgG class, IgE has several potential advantages over Abs of the IgG class, such as the IgE-FcεRI high affinity interaction, which allows a more effective arming of effector cells without losing surface-bound Abs (30, 70, 71) and the low serum levels of IgE that result in less competition for FcR occupancy (70–72). The first clinical trial (www.clinicaltrials.gov; clinical trial number NCT02546921) is currently underway in patients with advanced solid tumors to examine the safety of a mouse/human chimeric IgE Ab (MOv18 IgE), specific for the tumor-associated antigen folate receptor-α, which has exhibited superior anti-tumor efficacy for IgE compared with IgG1 in animal models (73, 74).

Three separate experimental approaches were used above to demonstrate ADMC, and mediators from FcεRI-challenged ADMC, have anti-tumor activity and suggests the possibility of using autologous (or allogeneic) MC in cancer immunotherapy. There are several advantages for this potential technology. First, mature, functional, autologous or allogeneic MC can be obtained in quantities necessary for patient infusion. Second, the availability of IgE Abs with human constant regions (chimeric, humanized, and fully human) targeting tumor antigens has grown substantially (70, 72). Third, the high affinity binding between IgE and FcεRI is very stable with a long half-life resulting in an effective arming of MC, which would be able to target the tumor and so doing induce tumor cell death. The presence of dead tumor cells would facilitate their uptake and presentation of tumor antigens by antigen presenting cells (APC), eliciting an adaptive broad-spectrum anti-tumor immunity. This would increase due to MC local release of GM-CSF (19, 20) and potentially the release of suppressors of regulatory T-cell (Tregs) function as reported for IgE degranulation in murine MC (75). Lastly, unlike other immune cells currently being used for cancer immunotherapy (76), ADMC sensitized with anti-HER2/*neu* IgE are equipped to kill tumor cells without genetic reprogramming, which is time consuming and expensive (76).

In conclusion, it is shown that adipose tissue represents an alternative source for human MC that are phenotypically and functionally similar to primary MC. This new source of MC, ADMC, can be used in research to address fundamental questions in MC biology and to study IgE Abs including those targeting tumor antigens. Importantly, ADMC exhibit tumoricidal activity when armed with IgE Abs specific for a tumor antigen. Future studies are needed to evaluate the utility of ADMC, sensitized with tumor targeting IgE, to examine anti-tumor activity and toxicity in *in vivo* cancer models to further validate this potential new cancer immunotherapy strategy.

## Author Contributions

JP, ME, and MF conducted the experiments which were conceived by CK. MP, and TD-W developed the anti-HER2/*neu* IgE antibodies and helped design the studies. JP, ME, TD-W, MP, AD, and CK assisted in the preparation of this manuscript.

### Conflict of Interest Statement

The authors declare that the research was conducted in the absence of any commercial or financial relationships that could be construed as a potential conflict of interest.

## Abbreviations

MC: mast cells
Abs: antibodies
ADMC: adipose-derived mast cells
TNF-α: tumor necrosis factor alpha
GM-CSF: granulocyte-macrophage colony-stimulating factor
SCF: stem cell factor
PI: propidium iodide
APC: antigen presenting cells
Tregs: Regulatory T cells
NS: non-specific.
